# Two Rare Human Mitofusin 2 Mutations Alter Mitochondrial Dynamics and Induce Retinal and Cardiac Pathology in *Drosophila*

**DOI:** 10.1371/journal.pone.0044296

**Published:** 2012-09-05

**Authors:** William H. Eschenbacher, Moshi Song, Yun Chen, Poonam Bhandari, Peter Zhao, Casey C. Jowdy, John T. Engelhard, Gerald W. Dorn

**Affiliations:** Center for Pharmacogenomics, Department of Internal Medicine, Washington University School of Medicine, St. Louis, Missouri, United States of America; University of Sheffield - MRC Centre for Developmental and Biomedical Genetics, United Kingdom

## Abstract

Mitochondrial fusion is essential to organelle homeostasis and organ health. Inexplicably, loss of function mutations of mitofusin 2 (Mfn2) specifically affect neurological tissue, causing Charcot Marie Tooth syndrome (CMT) and atypical optic atrophy. As CMT-linked Mfn2 mutations are predominantly within the GTPase domain, we postulated that Mfn2 mutations in other functional domains might affect non-neurological tissues. Here, we defined *in vitro* and *in vivo* consequences of rare human mutations in the poorly characterized Mfn2 HR1 domain. Human exome sequencing data identified 4 rare non-synonymous Mfn2 HR1 domain mutations, two bioinformatically predicted as damaging. Recombinant expression of these (Mfn2 M393I and R400Q) in Mfn2-null murine embryonic fibroblasts (MEFs) revealed incomplete rescue of characteristic mitochondrial fragmentation, compared to wild-type human Mfn2 (hMfn2); Mfn2 400Q uniquely induced mitochondrial fragmentation in normal MEFs. To compare Mfn2 mutation effects in neurological and non-neurological tissues *in vivo*, hMfn2 and the two mutants were expressed in *Drosophila* eyes or heart tubes made deficient in endogenous fly mitofusin (dMfn) through organ-specific RNAi expression. The two mutants induced similar *Drosophila* eye phenotypes: small eyes and an inability to rescue the eye pathology induced by suppression of dMfn. In contrast, Mfn2 400Q induced more severe cardiomyocyte mitochondrial fragmentation and cardiac phenotypes than Mfn2 393I, including heart tube dilation, depressed fractional shortening, and progressively impaired negative geotaxis. These data reveal a central functional role for Mfn2 HR1 domains, describe organ-specific effects of two Mfn2 HR1 mutations, and strongly support prospective studies of Mfn2 400Q in heritable human heart disease of unknown genetic etiology.

## Introduction

Cycles of mitochondrial fusion and fission are an essential component of the mitochondrial quality-control apparatus [1], [2]. Mitochondrial fusion is a multi-step process requiring sequential tethering of two organelles, fusion of their outer mitochondrial membranes (OMM), and then fusion of their inner mitochondrial membranes [3]. The initial two stages of mitochondrial fusion, tethering and OMM fusion, are mediated by mitofusin (Mfn) proteins [4]. These large (757 amino acid) evolutionarily conserved GTPases are embedded in OMM. When the cytosolic domains of two opposing Mfn molecules interact (as when two different mitochondria touch), they connect via their respective cytosolic second heptad repeat (HR2) domains (amino acids 694–739), tethering the two organelles [5]. Mfn2 GTPase activity is necessary for both mitochondrial tethering and OMM fusion [6], [7].

Mammals express two mitofusins, Mfn1 and Mfn2, which are largely redundant when mediating mitochondrial fusion [8]. Genetic ablation of either Mfn1 or Mfn2 in the mouse germ line produces embryonic lethality, revealing a poorly understood developmental function for mitofusins [9]. Tissue-specific ablation of either Mfn1 or Mfn2 alone has minimal effects, whereas combined ablation of both Mfn1 and Mfn2 in neurons and striated muscle induces mitochondrial fragmentation (from unopposed mitochondrial fission) and causes severe, often lethal, end-organ dysfunction [10], [11], [12].

Missense mutations of Mfn2 are the most common recognized genetic defect for the human neurodegenerative condition Charcot-Marie Tooth Syndrome type 2A (CMT2) [13], and are a rare cause of Optic Atrophy (OA) [14]. An autosomal dominant pattern of inheritance of most human diseases linked to Mfn2 mutations and the results of recombinant expression studies in tissue culture indicate that disease-causing Mfn2 mutants can act as dominant inhibitors, impairing fusion by normal Mfn1 or Mfn2. Although mitochondrial fusion is essential for normal mammalian heart function [12], primary cardiac involvement in CMT2 is inexplicably rare [15], [16]. Indeed, mitochondrial fusion and respiratory function are reportedly normal in primary fibroblasts derived from patients with different CMT2 Mfn2 mutations [17], [18]. These findings suggest that currently recognized disease-causing Mfn2 mutations have distinct organ-specific effects.

An increasing number of rare human sequence variations are being uncovered by large-scale whole-exon and whole-genome sequencing projects. Many of these rare mutations will be deleterious (i.e. subject to purifying selection) and/or damaging (i.e. predisposing to disease) [19]. Based on the recent discovery that the Mfn2 HR1 domain plays a crucial role in Mfn2 protein interactions [20], we hypothesized that rare mutations affecting Mfn2 HR1 might affect tissues in addition to or other than those affected by the CMT2 mutations. Accordingly, we searched current human mutation databases for potentially damaging Mfn2 HR1 mutations and evaluated their pathological potential in *Drosophila melanogaster*, focusing on the retina and heart tube wherein disruption of mitochondrial fusion is known to induce dysfunction [21], [22], [23]. Both Mfn2 HR1 mutations induced retinal and cardiac pathology, but the magnitude of the mutational effects differed between the two tissues and the specific manifestations of each mutation were distinct.

## Results

### Identification of Potentially Pathological Nonsynonymous Human Mfn2 HR1 Mutations

**Figure 1:**
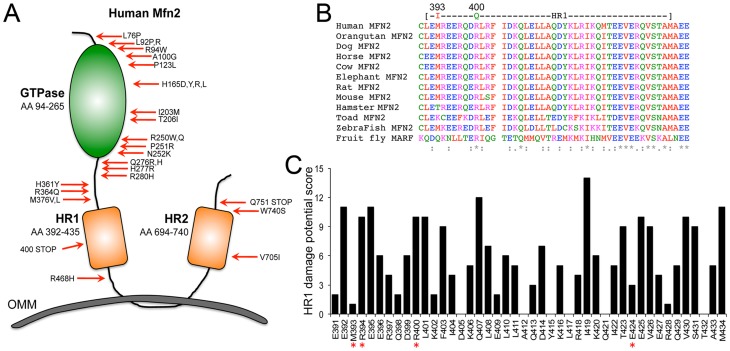
Characteristics of human Mfn2 mutations. (**A**) Schematic depiction of the locations for Mfn2 mutations linked with Charcot-Marie Tooth disease. HR = heptad repeat; OMM = outer mitochondrial membrane. (**B**) Multi-species sequence alignment for Mfn2 HR1 domain, indicating positions of potentially pathological amino acid 393 and 400 mutations. (**C**) Damage potential scoring for amino acids within the human Mfn2 HR1 region. Score is sum of Polyphen2 categorization for all possible non-synonymous changes at each nucleotide within a given codon; benign = 0, possibly damaging = 1, probably damaging or truncating = 2. Asterisks show amino acid positions of human mutations.

Most reported Mfn2 mutations in CMT syndrome [14], [24], [25], [26], [27] are clustered within its GTPase domain (**Figure 1a**). Bioinformatics analysis of 31 CMT-linked mutations by amino acid homology with SIFT [28] or multiple sequence- and structure-based features with Polyphen 2 [29] scored 84% as pathological; only two mutations (L76P and R468H) were predicted to be benign by both programs. Thus, available bioinformatics generally agree with genetic findings in predicting the pathology of human Mfn2 mutations.

A recent study uncovered unsuspected roles for the Mfn2 HR1 domain in intra- and inter-molecular interactions [20]. However, CMT-linked mutations are not found in the HR1 domain, except for a truncation mutation at amino acid 400 (**Figure 1a** and **Table S1**). Nevertheless, the Mfn2 HR1 domain shows a high degree of cross-species amino acid conservation, similar to the GTPase domain (**Figure 1b**). For this reason we hypothesized that rare naturally-occurring Mfn2 mutations within the HR1 domain might have pathological potential that, because of tissue-specific effects, have been overlooked in studies focused on CMT syndrome. We searched the NHLBI GO Exome Sequencing Project (ESP) database (NHLBI Exome Sequencing Project, Seattle, WA: http://evs.gs.washington.edu/EVS/) for rare non-synonymous sequence variants within the HR1 domain (i.e. amino acids 391–434, **Table S2**). Four non-synonymous HR1 sequence variants were identified: rs12069578 encoding M393I, rs186448929 encoding R394C, rs138072432 encoding R400Q, and rs8192303 encoding E424D. Bioinformatics analyses of these variants using SIFT and Polyphen2 [30] predicted that Mfn2 400Q and 393I might be damaging, whereas Mfn2 394C and 424D were predicted by both algorithms to be benign (**Table 1**).

**Table 1:** Characteristics of non-synonymous human Mfn2 HR1 mutations.

AA Pos	Ref AA	Mut AA	Allele frequency	SIFT score	SIFT Pred	Polyphen2 score	Polyphen2 Pred
393	M	I	0.0015	0.88	Tolerated	0.161	Poss Damag
394	R	C	NR	0.91	Tolerated	0.02	Benign
400	R	Q	0.0003	0.99	Damaging	0.999	Prob Damag
424	E	D	NR	0.92	Tolerated	0.001	Benign

SIFT score ranges from 0 to 1. The prediction based on a score larger than 0.95 is “Damaging”; otherwise, it is “Tolerated”. Polyphen2 score also ranges from 0 to 1. The prediction based on a score larger than 0.85 is “Probably damaging”; “Possibly damaging” if it is between 0.85 and 0.15 and “benign” if smaller than 0.15.

To better understand the pathological potential of Mfn2 HR1 mutations we performed an additional bioinformatics experiment. We considered that the precise consequences of any non-synonymous DNA nucleotide substitution are determined by the reference amino acid codon and the specific nucleotide change. We therefore used dbNSFP1.5 [30] to individually analyze each of the 263 possible non-synonymous DNA changes within the Mfn2 HR1 domain, estimating the damaging potential for all possible stochastic nucleotide sequence substitutions. SIFT and Polyphen2 predicted that 54% and 59% of all possible Mfn2 HR1 mutations would be possibly damaging (compared to 77% and 84% respectively of the 31 CMT-linked mutations; P = <0.001 for both [Fisher exact probability test]). Although the scales have different implications, a score closer to 1 is worse for both prediction systems; the mean SIFT and Polyphen2 scores for the all possible Mfn2 HR1 mutations were 0.836545±0.014996 and 0.467024±0.025554, respectively (compared to 0.942763±0.02600 and 0.718099±0.06803 for the 31 CMT-linked mutations; P = 0.0179 for SIFT and 0.0014 for Polyphen2 score [2-tailed student t test]). Thus, random nucleotide substitutions within the HR1 coding domain appear less likely to damage Mfn2 than known disease-related mutations. However, individual Mfn2 HR1 amino acid positions vary greatly in predicted pathological potential (**Figure 1c**): pathological potential at position 393 is low, with the rs12069578 M to I mutation the only possible variant scored other than benign. By contrast, pathological potential at position 394 is high, but the observed rs186448929 R to C mutation is the only possible substitution scored as not damaging. Every possible nucleotide substitution at amino acid position 400 was predicted to be highly damaging, including both the rs138072432 R to Q substitution mutation and the truncation mutation previous linked to CMT syndrome [14]. Finally, amino acid position 424 had a fairly low pathological potential, and the rs8192303 E to D substitution was predicted to be benign (**Figure 1c**).

### Recombinant Mfn2 393 and 400 Induce Mitochondrial Fragmentation in Murine Embryonic Fibroblasts

Bioinformatics analyses with SIFT and Polyphen2 agreed that the Mfn2 394C and 424D variations are benign substitutions. Therefore, our further biological evaluations focused on Mfn2 400Q scored as pathological by both SIFT (score of 0.99) and Polyphen2 (score of 0.999) and Mfn2 393I that SIFT classified as tolerated (score of 0.88) and Polyphen2 classified as possibly damaging (score of 0.161).

**Figure 2:**
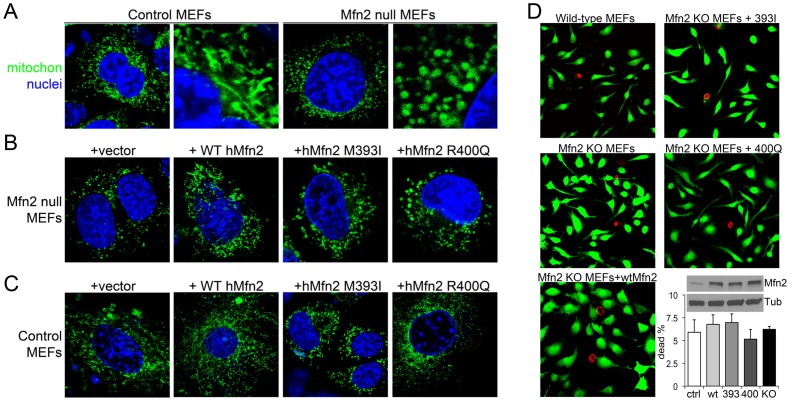
Human Mfn2 M393I and R400Q mutations are dysfunctional for inducing mitochondrial fusion in Mfn2-deficient MEFs. (**A**) Confocal analyses (600×) of MitoTracker green stained wild-type (left panels, Control) and Mfn2-null (right panels) MEFs. Nuclei are stained blue with DAPI. Right panels of each pair are enlarged sections of the left panel. Mfn2-null cells exhibit characteristic mitochondrial fragmentation. (**B**) MitoTracker green staining of Mfn2-null MEFs transfected with empty vector (first panel), wild-type human Mfn2 (WT hMfn2, second panel), the M393I human Mfn2 mutant (third panel), or the R400Q human Mfn2 mutant (fourth panel). Wild-type hMfn2 normalizes mitochondrial morphology in Mfn2-null MEFs, whereas phenotypic rescue by M393I is incomplete and R400Q Mfn2 has no effect. (**C**) As with (B), except performed in wild-type control MEFs. WT Mfn2 induces mitochondrial hyper-elongation, M393I has no significant effects, and R400Q induces mitochondrial fragmentation. (**D**) Representative images and quantification of live (green) and dead (red) MEFs and representative immunoblot of hMfn2 transgene expressions in MEFs.

Because Mfn2 is essential for normal mitochondrial fusion [8], our initial screens for dysfunctional human Mfn2 mutations assessed mitochondrial morphology in Mfn2-deficient murine embryonic fibroblasts (MEFs). As depicted in **Figure 2a** (left 2 panels), mitochondria in normal MEFs are elongated and appear interconnected within a web-like network. By comparison, mitochondria in Mfn2-deficient MEFs are individually distinct and more rounded (so-called “fragmented mitochondria”) (**Figure 2a**, **right 2 panels**). This is the morphological hallmark of defective mitochondrial fusion [8]. We assessed the efficiency of the mutant Mfn2s to induce mitochondrial fusion by attempting to rescue this “fragmented” mitochondrial phenotype. Compared to vector-transfected Mfn2 null MEFs, expression of wild-type hMfn2 eliminated mitochondrial fragmentation and normalized mitochondrial morphology (**Figure 2b** **, left 2 panels**). Expression of Mfn2 393I also improved mitochondrial morphology of Mfn2 null cells, although the mitochondria still appeared shorter and more rounded than normal (**Figure 2b** **, third panel**). Expression of Mfn2 400Q had no detectable benefit on Mfn2 null MEFs (**Figure 2b, right panel**).

We considered that the requirement for two interacting Mfn2 molecules to promote mitochondrial fusion is an ideal situation for dominant inhibition, even if only one member of the tethering pair is dysfunctional. We tested this notion by examining mitochondrial morphology in normal MEFs transfected with wild-type or the two hMfn2 mutants. As expected [31], expression of wild-type hMfn2 induced hyper-fusion of mitochondria, exaggerating the normal mitochondrial network (**Figure 2c** **, second panel**). Increased mitochondrial networking was not induced by Mfn2 393I, and in many cells the mitochondria appeared less connected and more rounded than normal (**Figure 2c** **, third panel**). Strikingly, expression of Mfn2 400Q was sufficient to induce severe mitochondrial fragmentation even in normal MEFs (**Figure 2c, right panel**). These findings reveal that the Mfn2 393I and 400Q exhibit varying degrees of dysfunction: Mfn2 393I is capable of mediating mitochondrial fusion in the absence of endogenous wild-type Mfn2, but appears less effective than wild-type Mfn2 for rescuing mitochondrial dysmorphology in Mfn2 null cells. In contrast, Mfn2 400Q lacks any intrinsic ability to induce mitochondrial fusion in Mfn2 null cells, and acts a potent dominant inhibitor of fusion induced by endogenous wild-type Mfn2.

Although mitofusin-deficient cells are viable [8], we determined if there was any relationship between disruption of Mfn-induced mitochondrial fusion and loss of cell viability using a live-dead fluorescence assay in which green calcein-AM stains live cells and red ethidium homodimer-1 stains dead cells. There were no differences in cell viability between Mfn2-expressing cells (**Figure 2d**). Immunoblot analysis showed that the Mfn2 mutants expressed at approximately the same levels as wild-type Mfn2 in transfected MEFs (**Figure 2d** **, right lower panel)**.

### *Drosophila* Eye Developmental Phenotypes Induced by Human Mfn2 393 and 400 Mutants

**Figure 3:**
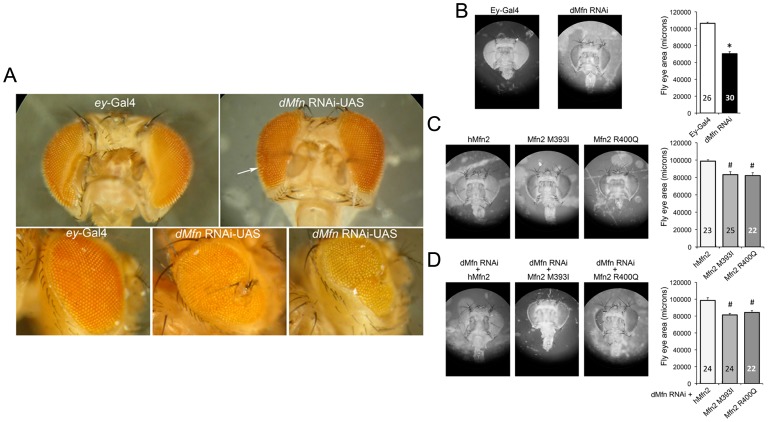
Human Mfn2 M393I and R400Q induce *Drosophila* eye abnormalities similar to eye-specific suppression of endogenous Drosophila mitofusin (dMfn). (**A**) Bright field microscopy images of adult control (ey-Gal4) and *Drosophila* mitofusin (dMfn) deficient (dMfn/MARF RNAi) eyes. dMfn2-deficient eyes are characteristically smaller and exhibit ommatidial disorganization and dysmorphology (white arrow). (**B-D**) Representative images and mean eye size data for *Drosophila* with eye-specific expression of wild-type or mutant human Mfn2, alone (**C**) or in the presence of dMfn RNAi (**D**) Eye size data are presented as mean ± SEM. Asterisk = p<0.05 vs Ey-Gal4. Pound = p<0.05 vs hMfn2 transgene in the same genetic background.

The *Drosophila* eye is dispensable for reproduction and development, and is therefore a useful organ in which the consequences of potentially lethal genetic manipulations can be interrogated *in vivo* [32], [33], [34]. Previously, decreased *Drosophila* eye size and/or roughening of the normal eye surface were observed with genetic manipulation of other members of the mitochondrial fusion/fission and quality control apparati [21], [22], [35]. Here, we observed a ∼30% reduction in eye area of *Drosophila* in which mitochondrial fusion was impaired through eye-specific expression of previously validated RNAi against the *Drosophila* Mfn ortholog dMfn/MARF [23], [36] (**Figure 3a, 3b**). This finding demonstrates that suppression of OMM fusion can also provoke eye phenotypes in *Drosophila*. Accordingly, we generated transgenic fly lines with eye-specific expression of wild-type hMfn2, Mfn2 393I, and Mfn2 400Q and determined their effects on eye morphology. Compared to control flies carrying only the Ey-Gal4 driver, expression of wild-type hMfn2 had no effect on *Drosophila* eye area (**Figure 3c**). By contrast, eye areas were significantly reduced in flies expressing either the Mfn2 393I or Mfn2 400Q mutant; the reduction in eye size induced by the hMfn2 mutants was approximately two thirds that induced by RNAi-mediated dMfn/MARF suppression (compare **Figures 3b** and 3c). The small eye phenotype was completely rescued by co-expressing wild-type hMfn2, but not by either of the mutants.

### Human Mfn2 393I and 400Q Mutants Induce Progressive Dilated Cardiomyopathy in *Drosophila*

**Figure 4:**
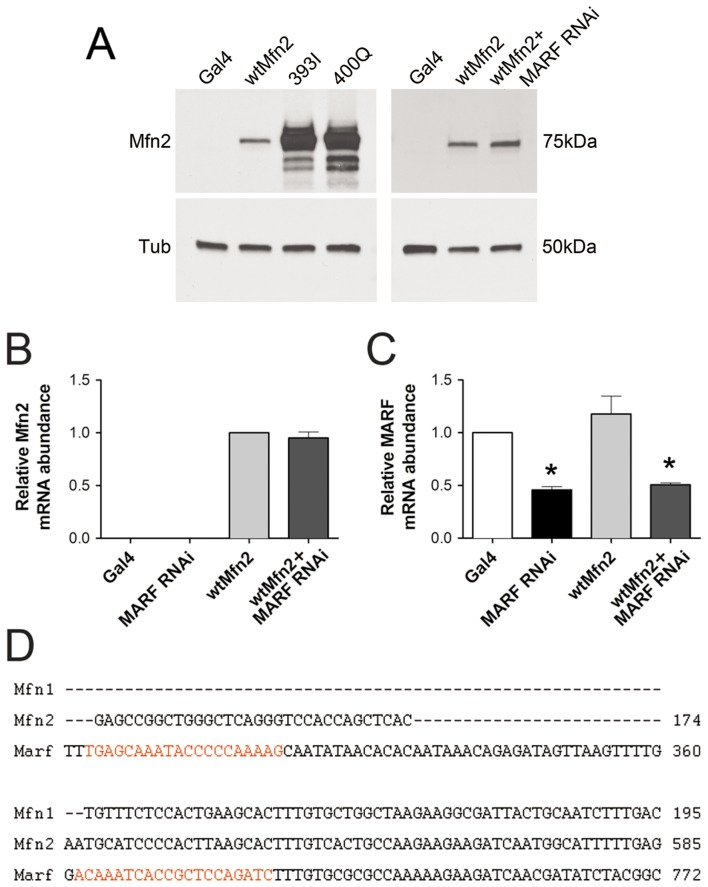
Expression of wild-type and mutant human Mfn2s. (**A**) The protein expression levels of hMfn2 in the heart tubes of wild-type and mutant hMfn2 transgenic flies and that in the presence of dMfn RNAi. (**B-C**) The mRNA levels of hMfn2 (B) and Marf (C) in the heart tubes of wild-type hMfn2 transgenic flies with or without dMfn RNAi. (**D**) Sequence alignment of human mitofusins and *Drosophila* Mfn. The sequences targeted by dMfn RNAi is marked in red.

*Drosophila* eye phenotypes are developmental in nature and static in manifestation. To explore the pathological potential of Mfn2 393I and 400Q on functioning of a dynamic organ we determined their effects on the heart tube, in which normal mitochondrial fusion is indispensable and it is possible to assess phenotypic progression over time. We previously described *Drosophila* heart tube dilation and impaired contraction after cardiomyocyte-specific suppression of dMfn/MARF or Opa1 [23]. In that work, expression of normal human Mfn2 rescued the cardiomyopathy induced by cardiomyocyte-specific RNAi suppression of *Drosophila* MARF [23]. Here, we crossed the previously described wild-type hMfn2 and newly developed mutant hMfn2 transgenic fly lines onto the tincΔ4-Gal4 driver to induce cardiomyocyte-specific expression [37]. As with the fly eye studies (above), wild-type and mutant hMfn2 were expressed both in normal cardiomyocytes or along with RNAi-mediated suppression of dMfn/MARF. Immunoblot analysis showed that mutant hMfn2 393I and 400Q were expressed in Drosophila heart tubes at equivalent levels, although expression was substantially greater than wild-type hMfn2 (**Figure 4a**). Furthermore, we demonstrated that the *Drosophila* dMfn/MARF RNAi did not affect transgenic expression of hMfn2 protein (**Figure 4b**) or mRNA (**Figure 4c**), consistent with nucleotide sequence divergence at the dMfn RNAi targeting regions (**Figure 4d**).

**Figure 5:**
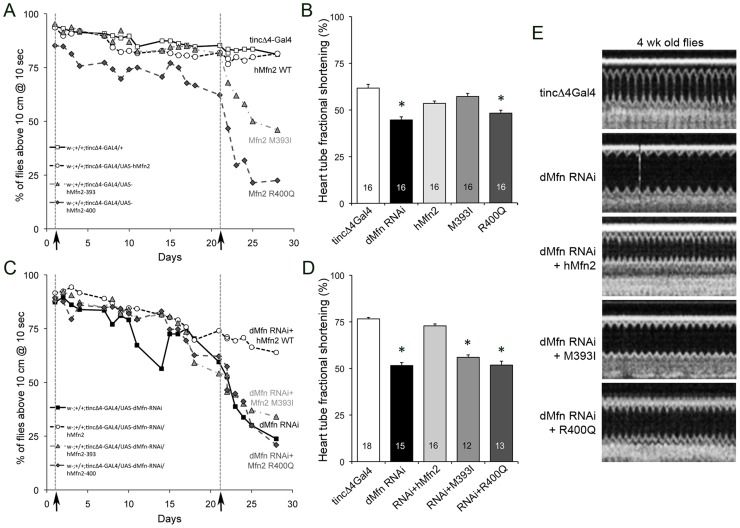
Human Mfn2 M393I and R400Q induce *Drosophila* cardiomyopathy/heart failure phenotypes recapitulating those caused by suppression of mitochondrial fusion. (**A, B**) Negative geotaxis exercise capacity (A) and optical coherence tomography (OCT) of heart tube fractional shortening (B) of flies deficient in endogenous mitofusin (dMfn RNAi) or expressing wild-type, M393I, or R400Q mutant human Mfn2. Dotted lines and arrows indicate time of OCT study. R400Q induces cardiomyopathy by 21 days, whereas M393I effects are less severe at that time. (**C, D**) As in A and B, except the wild-type and mutant human Mfn2s are expressed in mitofusin-deficient *Drosophila* heart tubes. Wild-type hMfn2 almost completely rescues loss of exercise capacity (C) and depressed heart tube contraction (D), whereas M393I and R400Q Mfn2 are similar to dMfn2 RNAi alone. OCT data are presented as mean ± SEM. Asterisk = p<0.05 vs tincΔ4-Gal4. (**E**) Representative OCT images from (D).

Our initial cardiac function studies examined negative geotaxis (the normal drive for *Drosophila* to climb up the sides of a container when dislodged to its bottom), which parallels cardiac performance in ageing *Drosophila* [38], [39]. At the beginning of the geotaxis study (1 week after eclosure) the rate of climbing was the same in control (tincΔ4-Gal4), wild-type hMfn2, and mutant Mfn2-expressing flies (**Figure 5a**, day 1). Daily climbing ability remained normal throughout the four-week experiment for cardiac wild-type hMfn2 flies (**Figure 5a**, day 28). However, approximately three weeks into the study climbing rates of the cardiac Mfn2 400Q mutant expressing flies diverged from controls. At the end of the four-week study cardiac Mfn2 400Q mutant flies were largely earth-bound, and cardiac Mfn2 393I flies exhibited diminished climbing (P<0.01 vs hMfn2 WT and Mfn2 400Q; ANOVA of repeated measures).

To relate exercise capacity with cardiac function in the various Mfn transgenic fly lines, we used optical coherence tomography (OCT) to measure heart tube contraction (assessed as % fractional shortening, %FS). OCT was performed 1 and 4 weeks after eclosure, corresponding to the beginning of the negative geotaxis studies, and the time of initial divergence of geotaxis response by the Mfn2 400Q flies (i.e. 3 weeks into the negative geotaxis experiment; **Figure 5a**, arrows and dashed lines). Flies carrying only the heart specific tincΔ4-Gal4 driver were studied as normal controls. Flies expressing wild-type and mutant human Mfn2 had a slightly decreasing heart tube fractional shortening at 1 week (**Figure S1a)**. In contrast, heart tube fractional shortening was significantly and uniquely depressed in 4-week-old *Drosophila* expressing Mfn2 400Q (**Figure 5b**). Thus, diminished exercise capacity and heart tube contractile dysfunction were concordant, and suggested a dominant negative effect of the 400Q Mfn2 mutant.

We also performed a “replacement rescue study” with the wild-type and mutant human Mfn2 flies on the dMfn/MARF RNAi background. RNAi-mediated suppression of cardiomyocyte dMfn profoundly impaired negative geotaxis as a function of time (**Figure 5c**), which was almost completely rescued by expressing wild-type hMfn2 (**Figure 5c**). By comparison, neither the 400Q nor the 393I Mfn2 mutant rescued the decreased exercise capacity resulting from suppression of dMfn (**Figure 5c**), even though they are expressed at higher levels than wild-type hMfn2. In parallel OCT studies, cardiomyocyte-specific suppression of dMfn depressed heart tube contractions (compared to tincΔ4-Gal4 controls); this cardiomyopathy was rescued by concurrent expression of wild-type Mfn2, but not mutant M393I or R400Q Mfn2 at both 1 and 4 weeks (**Figures 5d, 5e** and **Figure S1b**).

### Mfn2 HR1 Mutants Induce Mitochondrial Fragmentation in Cardiac Myocytes

**Figure 6:**
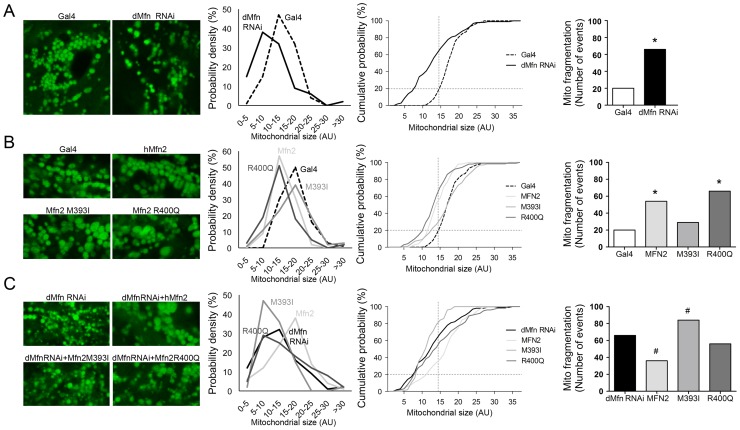
Cardiomyocyte mitochondrial fragmentation parallels *Drosophila* cardiomyopathy induced by human Mfn2 mutants. (**A–C**) *Drosophila* cardiomyocyte mitochondria visualized with tincΔ4-Gal4-driven mito-GFP in control (Gal4) and transgenic fly lines corresponding to those in Figure 5. Mitochondrial population size is quantified by measuring the diameter of individual mitochondria, presented in probability density curve (left graph), cumulative distribution curve (middle graph) and comparison of fragmented mitochondrial populations (right graph). Suppression of endogenous cardiomyocyte dMfn with the RNAi induces mitochondrial fragmentation, shown as smaller organelles and a leftward shift in the size distribution (A) Increased mitochondrial heterogeneity and/or fragmentation is seen with expression of wild-type or mutant human Mfn2 in the presence of endogenous dMfn (B). In dMfn-deficient heart tubes (C), wild-type human Mfn2 normalizes mitochondrial size, whereas mitochondrial fragmentation persists after Mfn2 M393I or R400Q replacement. Asterisk = p<0.05 vs tincΔ4-Gal4. Pound = p<0.05 vs dMfn RNAi.

Taken together, the above results reveal that the rare human Mfn2 M393I and R400Q mutants confer similar *Drosophila* developmental eye phenotypes, but differ in their capacity to: 1. Induce mitochondrial fragmentation in normal cultured MEFs; 2. Rescue mitochondrial fragmentation in Mfn2-deficient MEFs; and 3. Confer *Drosophila* cardiac phenotypes resembling those induced by suppression of *Drosophila* Mfn. To define the mechanism for functional distinctiveness of the two human Mfn2 mutants in the *Drosophila* heart tube, we examined cardiomyocyte mitochondrial morphology in flies expressing wild-type and mutant Mfn2, with or without suppression of endogenous dMfn. As previously described [23], confocal microscopy of normal *Drosophila* cardiomyocyte mitochondria visualized using tincΔ4-Gal4 driven mitochondrial-targeted green fluorescent protein reveals a relatively homogenous population of rounded organelles (**Figure 6a**). Suppression of cardiomyocyte mitochondrial fusion with dMfn RNAi increased mitochondrial heterogeneity and induced organelle fragmentation. Quantitative analysis of mitochondrial dimension revealed a shift in the population distribution toward smaller organelles in dMfn RNAi expressing cardiomyocytes. Consistent with the results of the negative geotaxis and heart tube contraction studies (vide supra), expression of the M393I Mfn2 mutant increased mitochondrial heterogeneity compared to tincΔ4-Gal4 controls (**Figure 6b**, contrast the width of the probability distribution curves). By comparison, expression of the 400Q Mfn2 mutant increased both mitochondrial heterogeneity and fragmentation defined as the proportion of mitochondria in the lowest quintile of normal size; these effects were not as severe as with suppression of *Drosophila* dMfn with the RNAi (**Figure 6a** **, right two images**).

As it did in the negative geotaxis and cardiac contraction studies, substitution of *Drosophila* dMfn/MARF with either of the human Mfn2 mutants provoked more severe mitochondrial dysmorphology than superimprosed expression (**Figure 6c**). Mitochondrial fragmentation induced by dMfn RNAi was almost completely normalized by wild-type human Mfn2. By contrast, dMfn RNAi-induced mitochondrial fragmentation persisted after attempted “rescue” with human Mfn2 M393I or R400Q (**Figure 6c** **, right images**). We also noted that the Mfn2 400Q mutant produced occasional abnormally large mitochondria. It has been speculated that sporadic cardiomyocyte mitochondrial enlargement accompanying defective mitochondrial fusion is the consequence of impaired mitochondrial clearance [12].

## Discussion

These findings reveal that the heretofore largely overlooked HR1 region of Mfn2 is a functionally important domain sensitive to naturally occurring mutations. We describe organelle, organ, and organism dysfunction induced by two recently discovered human mutations of Mfn2, Mfn2 M393I (rs12069578) and Mfn2 R400Q (rs138072432). Both of these Mfn2 mutations are quite rare in the populations that have undergone whole-exome sequencing (reported allele frequencies of 0.002 and 0.001, respectively) and were classified as potentially damaging by some bioinformatic algorithms. Our functional studies show that Mfn2 393I is intrinsically dysfunctional, but has little effect when expressed along with normal Mfn. We expect that this mutation would therefore not be sufficient to cause disease in the heterozygous state. Strikingly, Mfn2 400Q not only is incapable of promoting mitochondrial fusion on its own, but it acts as a potent dominant inhibitor of mitochondrial fusion in MEFs and *Drosophila* heart tubes having a normal complement of endogenous mitofusin (i.e. *Drosophila* Mfn/MARF and MEF Mfn1 and Mfn2). The implication of this finding is that Mfn2 R400Q could induce pathology even in individuals who are heterozygous for the mutation.

The impetus for these studies was our recent observation that genetic loss-of-function of mitochondrial fusion proteins is sufficient to induce cardiomyopathy phenotypes in murine and *Drosophila* models [12], [23]. We infer from these findings that mitochondrial fusion is essential to normal cardiac function. In the human condition, however, loss-of-function Mfn2 mutations identified as causing neurodegenerative syndromes have not been associated with cardiac disease. Instead, mutations clustered predominantly in the Mfn2 GTPase domain cause hereditary Charcot Marie Tooth syndrome type 2a and atypical forms of optic atrophy [13], [14]. We considered that mutations outside the GTPase domain might preferentially affect other organs/organ systems. Accordingly, we undertook the first functional studies of rare (and therefore potential pathological; [19]) naturally occurring mutations within the human Mfn2 HR1 domain. By comparing the effects of the 393I and 400Q mutations in a neurological tissue, the *Drosophila* eye, and the heart tube, our studies uncovered *in vivo* tissue-specific differences in mutational effects.

The Mfn2 first heptad repeat (HR1) (amino acids 392–435) is highly conserved throughout vertebrate evolution. This region of the protein has been considered less important for mitochondrial fusion than the GTPase and HR2 domains [40]. However, recent studies have uncovered a central role for HR1 in intra-molecular Mfn2 binding (with HR2) and in inter-molecular interactions with the mitochondrial fission protein, dynamin-like protein 1 (Dlp1, previously known as Drp-1) [20]. Bioinformatically predicted pathological human Mfn2 HR1 mutations have only recently been described through large-scale exon-sequencing and the numbers of affected individuals are too small to make any statistical genetic linkage with disease. Thus, the current findings are valuable by establishing that these two Mfn2 HR1 mutations are indeed dysfunctional. These data further reveal that the consequences of the Mfn2 393I and 400Q mutations differ depending upon physiological context, i.e. tissue-type and the presence of normal mitofusins. Even in the presence of endogenous mammalian Mfn2 or *Drosophila* dMfn, the 400Q mutant that was bioinformatically predicted to be the more pathological of the two mutants induced mitochondrial dysmorphology and conferred a severe cardiomyopathy phenotype, demonstrating dominant inhibition of endogenous mammalian and invertebrate Mfns by Mfn2 400Q. Under the same conditions Mfn2 393I had little effect on mitochondrial morphology in MEFs or *Drosophila* cardiomyocytes, did not impair heart tube contractile function, and induced only modest depression of exercise capacity measured by the negative geotaxis response. However, Mfn2 393I does not function as a normal Mfn2 because (in contrast to wild-type Mfn2 expressed in MEFs at similar levels or in the *Drosophila* heart at far lower levels) it was not capable of rescuing the mitochondrial and functional phenotypes of Mfn2 null MEFs or dMfn deficient *Drosophila* heart tubes. Indeed, in the context of intrinsic Mfn insufficiency, the phenotypes conferred by Mfn2 393I and 400Q were almost equally severe. Accordingly, we conclude that Mfn2 393I is a functional null with little dominant inhibitor activity.

It is worth emphasizing that the consequences of the Mfn2 M393I and R400Q substitutions were very different in the *Drosophila* eye. First, the human Mfn2 mutants decreased eye size to the same extent in *Drosophila* eyes. Second, the severity of eye pathology conferred by the two human Mfn2 mutants was not affected by the presence or absence of endogenous *Drosophila* dMfn2. Third, in contrast to their effects in the *Drosophila* heart tube, the extent of the eye abnormality conferred by the mutants was only about 50% as severe as that induced by RNAi-mediated suppression of endogenous dMfn. We postulate that the different manifestations of Mfn2 HR1 mutants in Drosophila eye and heart tube may relate on one hand to the static nature of the adult *Drosophila* eye and on the other to the relative dispensability of heart tube function in the *Drosophila* embryo. Thus, *Drosophila* eye phenotypes reflect almost entirely processes that are affected during embryonic development whereas *Drosophila* heart tube phenotypes are manifested almost entirely after the adult fly emerges.

Our study is somewhat limited in that the relevance of mitofusin function in the *Drosophila* heart or eye to the human condition remains to be established. Importantly, *Drosophila* express only one mitofusin protein (dMfn/Marf) in their hearts whereas mammals express both Mfn1 and Mfn2, with largely overlapping functions for mitochondrial fusion. Thus, simple loss of function without dominant inhibition that is exhibited by Mfn2 393I is likely to have little effect on mammalian hearts, because Mfn1 can substitute. On the other hand, if our idea is correct that the dominant inhibitory effect of Mfn2 400Q relates to its disruption of both homotypic (i.e. Mfn2-Mfn2) and heterotypic (i.e. Mfn1-Mfn2) mitofusin interactions, then its pathological potential should be equally great in mammalian heart disease. Clearly additional work is warranted to define the precise molecular mechanism for dysfunction induced by these two HR1 Mfn2 mutations and to examine their consequences in mammalian hearts. Finally, the unexplained difference in protein expression levels between our old wild-type hMfn2 fly line [23] and the new 393I and 400Q mutant hMfn2 fly lines may appear to be an additional confounder for the fly heart studies (levels were equivalent in the MEF studies). However, there is no evidence that increased Mfn2 expression has any toxic effects in any system. Therefore, phenotypic rescue by lower levels of wild-type hMfn2, but not by the mutants, only further emphasizes their dysfunction.

Notwithstanding the anticipated availability of entire human genome sequence for $1,000, the evaluation of rare alleles as causative factors or modifiers of human disease is unlikely to be achieved through large-scale whole genome (or exome) population sequencing projects. Discrete tissue-specific phenotypes are unlikely to be adequately described in massive population studies and rare human genetic diseases will be under-represented. Thus, a purely unbiased genetic epidemiology approach linking rare mutations to human disease will be limited by suboptimal sampling and statistical power. Indeed, no phenotypic information is available for any of the four human Mfn2 HR1 mutants described herein, and it appears that Mfn2 393I and 400Q have each only been detected in one or two individuals. Even when 1,000 (or 10,000 or 1,000,000) whole genomes data become available and mutations are prioritized by bioinformatic predictions of pathological potential, definitive human investigations will need to be guided by assessments of functional activity and phenotypic manifestation in intact *in vivo* biological systems. Our findings demonstrate the utility of *Drosophila* for this type of rapid functional stratification of suitable rare human mutations. We believe that these data establish a powerful rationale for targeted genetic discovery and linkage analysis of Mfn2 HR1 mutations in heritable human cardiomyopathies with an unidentified genetic component.

## Materials and Methods

### Bioinformatic Identification of Human Mfn2 Gene Variants

Mfn2 mutation data were obtained from the NHLBI GO Exome Sequencing Project [41]. *In silico* characterization of pathological potential for the four non-synonymous human Mfn2 HR1 domain mutations, for published Charcot-Marie Tooth syndrome Mfn2 mutations, and for all possible non-synonymous mutations of the Mfn2 HR1 domain used SIFT and PolyPhen2 scoring from dbNSFP1.3 [30]. A damage potential score for each amino acid within the Mfn2 HR1 domain was calculated as the sum of the PolyPhen2 functional categorization for all possible non-synonymous substitutions at that position, where Benign was assigned a value of 0, Possibly Damaging = 1, and Probably damaging or truncation mutation = 2.

### Cell Lines and Confocal Analyses of Mitochondria

Wild-type MEFs were prepared from ∼E16.5 mouse embryos. Mfn2-null MEFs were obtained from ATCC, Manassas, VA (#CRL-2993). The M393I and R400Q mutations were introduced into wild-type human Mfn2 cDNA through PCR mutagenesis and confirmed by full-length sequencing. Wild-type and mutant hMfn2 were expressed in MEFs after subcloning into pcDNA3 (Invitrogen). Mitochondria were stained with 100 nM MitoTracker GreenFM (Invitrogen, Carlsbad, CA) for 30 minutes and mounted using Vectashield containg DAPI (Vector, Burlingame, CA) for nuclear counterstaining. Cell viability was analyzed using Live/dead viability/cytotoxicity kit for mammalian cells (Invitrogen). Images were obtained on a Nikon C1si D- eclipse confocal microscope system and camera (Nikon Instruments, Melville, NY) using a Nikon Plan Apo VC 60 ×/1.40 oil objective and 6× digital zoom. The expression levels of hMfn2 transgenes were analyzed by protein electrophoresis and western blot using the same antibodies as stated below.

### *Drosophila* Stocks and Transgenic Lines

Fly stocks for UAS-mitoGFP (#8442) and ey3.5Gal4 (#8221) were obtained from the Bloomington *Drosophila* Stock Center at Indiana University. Rolf Bodmer (Sanford-Burnham Medical Research Institute, La Jolla, California) provided tincΔ4Gal4 Ming Guo (University of California, Los Angeles) provided dMFN/MARF RNAi-UAS; the sequence of the oligonucleotide targeting Marf UTR region is AGTCTAGATGAGCAAATACCCCCAAAAG and that targeting Marf coding region is AAGAATTCGATCTGGAGCGGTGATTTGT. Wild-type hMfn2 transgenic flies were constructed by subcloning the cDNA into pUAST as previously described [23]. M393I and R400Q Mfn2 mutant lines were created in the same manner. Three independent lines of wild-type and each mutant Mfn2 were initially characterized using the *ey* or *tinc* drivers, in the presence and absence of the dMfn/MARF RNAi.

### mRNA Expression Profiling

For RT-PCR, RNA was prepared from the heart tissue of 40–60 adult flies using RNA-Bee (Amsbio LLC) and cDNA was prepared by random priming and reverse transcription. PCR was used the following primers: hMfn2 Forward ATGCATCCCCACTTAAGCAC, Reverse AGCACCTCACTGATGCCTCT; MARF Forward GACAAATCACCGCTCCAGAT, Reverse GAAGGCCACCTTCATGTGAT, and quantified by real-time qPCR.

### Protein Electrophoresis and Western Blot Analysis

To analyze transgenic hMfn2 expression in *Drosophila* heart tubes, specimens were collected and protein was extract with Laemmli sample buffer (Bio-rad) containing 200 mM β-mercaptoethanol by boiling at 100°C for 10 min. After brief centrifugation to remove insoluble debris, extracted samples from 10 pieces of heart tubes for each *Drosophila* line were separated by 4–15% Mini-protean TGX gel (Bio-rad) and immunoblot was carried out using primary mouse-anti Mfn2 antibody (1∶1000, Abcam 56889) and mouse-anti α-tubulin antibody (1∶10,000, Sigma T6074) and secondary horse-anti-mouse-IgG (H&L) antibody (1∶4000, Cell signaling 7076). Each antibody was incubated with the membrane for 1 hr at room temperature.

### *Drosophila* Eye Studies

*Drosophila* heads were optically imaged in the frontal plane or in profile using a Nikon SMZ 1500 microscope with a HR Plan Apo 1× WD 54 objective at 112.5× magnification; images were archived with a Nikon Coolpix 5000 digital camera. Eye size was determined manually using a stage micrometer accurate to 10 µm; vertical measurements were taken from the dorsal most point of the eye to the ventral portion and horizontal measurements from the middle of the eye from medial to lateral.

### Negative Geotaxis Studies

About 150 newly emerged adult *Drosophila* of a given genotype were transferred into a ∼1 cubic foot ventilated plexiglass container and maintained there for the duration of the 4-week study. Beginning 1 week thereafter, twice daily the *Drosophila* were suddenly displaced to the bottom of the container and the number of flies that had climbed greater than 10 cm after 10 seconds was determined. The study continued for 4 weeks.

### *Drosophila* Heart Studies

Contracting *Drosophila* heart tubes of 1- and 4-week-old adult flies were imaged *in situ* using optical coherence tomography (OCT) [42], [43] on a Michelson Diagnostics (Maidstone, UK) EX 1301 OCT microscope at 1,300-nm wavelength laser directed transversely as previously described [23]. B-mode images were generated by post-analysis of 2-dimensional images. Internal chamber diameter at end systole (ESD) and end-diastole (EDD) were measured and % fractional shortening calculated as (EDD-ESD)/EDD.

Cardiomyocyte mitochondria were imaged in MitoGFP expressing heart tubes as described [23] on the Nikon Eclipse confocal system described above. Mitochondrial dimension was measured manually on high-resolution images.

### Statistical Methods

Groups were compared by student t-test or ANOVA with Bonferroni correction, as appropriate. To compare cardiomyocyte mitochondrial “fragmentation” across the different genetic groups, the lowest size quintile defined by the control (Gal4) group was defined and the proportion of mitochondria within this size range was compared using Fisher’s exact test. Significance was defined at P<0.05.

## Supporting Information

Figure S1 **OCT of heart tube fractional shortening of 1-week-old flies.** (**A**) Flies expressing wild-type and mutant human Mfn2 in the heart tubes had a slightly decreasing fractional shortening. (**B**) Flies expressing wild-type hMfn2 in dMfn-deficient heart tubes showed completely rescued contraction whereas M393I and R400Q Mfn2 were similar to dMfn2 RNAi alone. OCT data are presented as mean ± SEM. Asterisk = p<0.05 vs tincΔ4-Gal4.
(TIF)

Table S1 **Characteristics of human Charcot Marie Tooth Mfn2 mutations.**
(DOCX)

Table S2 **Pathological potential of possible HR1 amino acid mutations.**
(DOCX)
