# Supplementary figures and images for: Two Rare Human Mitofusin 2 Mutations Alter Mitochondrial Dynamics and Induce Retinal and Cardiac Pathology in Drosophila
Source: PLoS One. 2012 Sep 5;7(9):e44296. doi: 10.1371/journal.pone.0044296 (PMC3434137; doi:10.1371/journal.pone.0044296)

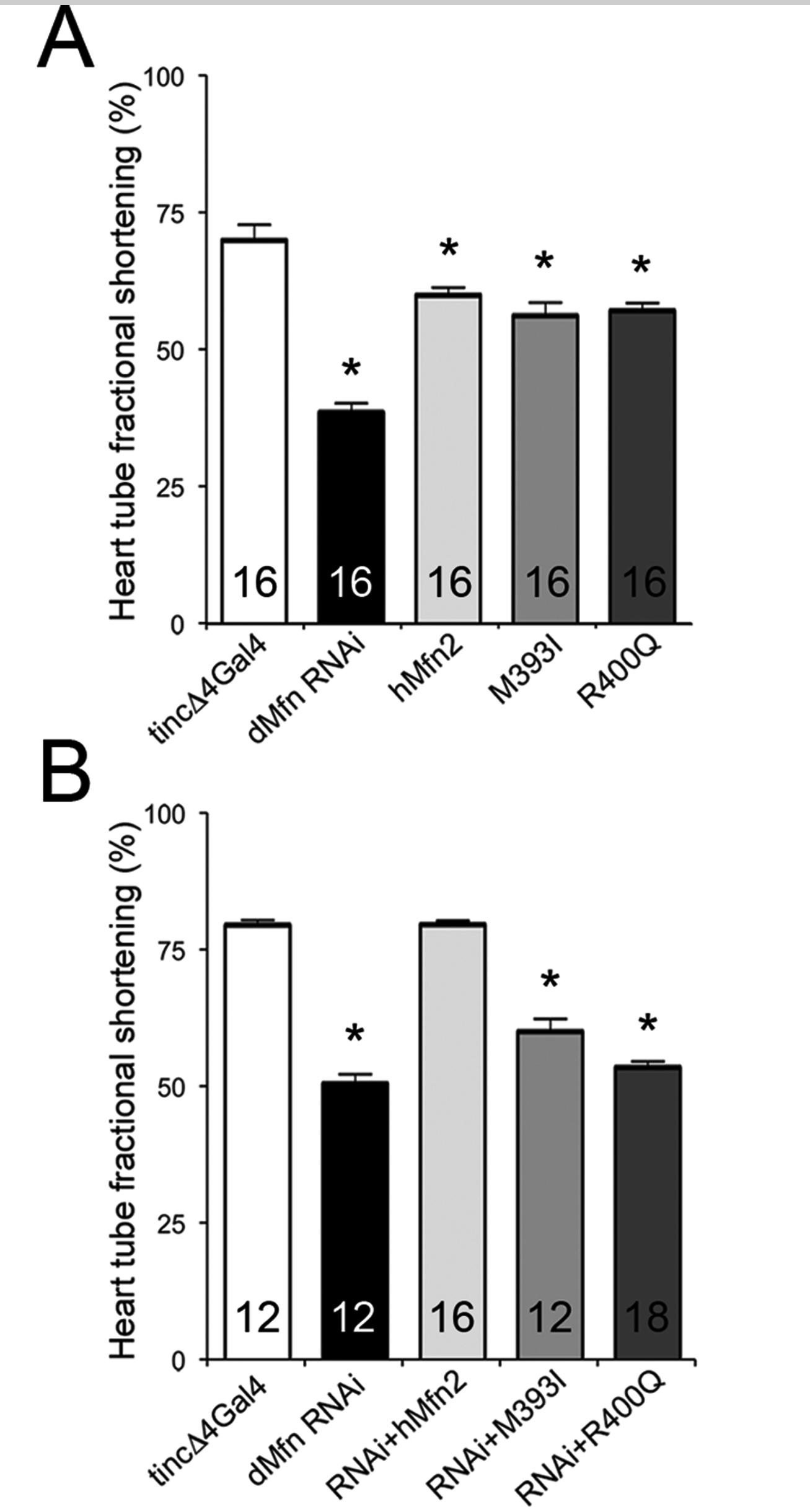

Supplement: Figure S1 — OCT of heart tube fractional shortening of 1-week-old flies. (A) Flies expressing wild-type and mutant human Mfn2 in the heart tubes had a slightly decreasing fractional shortening. (B) Flies expressing wild-type hMfn2 in dMfn-deficient heart tubes showed completely rescued contraction whereas M393I and R400Q Mfn2 were similar to dMfn2 RNAi alone. OCT data are presented as mean ± SEM. Asterisk = p<0.05 vs tincΔ4-Gal4. (TIF) [file pone.0044296.s001.tif]
