# Supplementary material for: Two Rare Human Mitofusin 2 Mutations Alter Mitochondrial Dynamics and Induce Retinal and Cardiac Pathology in Drosophila
Source: PLoS One. 2012 Sep 5;7(9):e44296. doi: 10.1371/journal.pone.0044296 (PMC3434137; doi:10.1371/journal.pone.0044296)
Supplement: Table S1 — Characteristics of human Charcot Marie Tooth Mfn2 mutations. (DOCX) [file pone.0044296.s002.docx]

**Table S1.** *Characteristics of human Charcot Marie Tooth Mfn2 mutations.*

| **Ref AA** | **Mut AA** | **AA position** | **SIFT_score** | **SIFT_pred** | **Polyphen2**  **score** | **Polyphen2**  **pred** |
| --- | --- | --- | --- | --- | --- | --- |
|  |  |  |  |  |  |  |
| L | P | 76 | 0.83 | Tolerated | 0.024 | Benign |
| L | P | 92 | 1 | Damaging | 0.956 | Prob Damag |
| L | R | 92 | 1 | Damaging | 0.077 | Benign |
| R | W | 94 | 1 | Damaging | 1 | Prob Damag |
| R | Q | 94 | 1 | Damaging | 0.999 | Prob Damag |
| A | G | 100 | 0.99 | Damaging | 0.012 | Benign |
| P | L | 123 | 1 | Damaging | 1 | Prob Damag |
| H | D | 165 | 0.94 | Tolerated | 0.999 | Prob Damag |
| H | Y | 165 | 0.99 | Damaging | 1 | Prob Damag |
| H | R | 165 | 0.99 | Damaging | 1 | Prob Damag |
| H | L | 165 | 1 | Damaging | 1 | Prob Damag |
| I | M | 203 | 0.98 | Damaging | 0.152 | Poss Damag |
| T | I | 206 | 1 | Damaging | 0.41 | Poss Damag |
| R | W | 250 | 1 | Damaging | 0.967 | Prob Damag |
| R | Q | 250 | 0.99 | Damaging | 0.376 | Poss Damag |
| P | R | 251 | 1 | Damaging | 1 | Prob Damag |
| N | K | 252 | 1 | Damaging | 0.999 | Prob Damag |
| Q | R | 276 | 1 | Damaging | 0.77 | Poss Damag |
| Q | H | 276 | 1 | Damaging | 0.879 | Prob Damag |
| H | R | 277 | 1 | Damaging | 0.999 | Prob Damag |
| R | H | 280 | 1 | Damaging | 0.999 | Prob Damag |
| H | Y | 361 | 1 | Damaging | 0.999 | Prob Damag |
| R | Q | 364 | 0.98 | Damaging | 0.823 | Poss Damag |
| M | V | 376 | 0.97 | Damaging | 0.974 | Prob Damag |
| M | L | 376 | 0.23 | Tolerated | 0.338 | Poss Damag |
| M | I | 376 | 0.95 | Tolerated | 0.974 | Prob Damag |
| R | X | 400 | 0.897625 | Truncation | 0.722706 | Truncation |
| R | H | 468 | 0.88 | Tolerated | 0.057 | Benign |
| V | I | 705 | 0.745987 | NA | 0.024 | Benign |
| W | S | 740 | 0.958256 | NA | 0.996 | Prob Damag |
| Q | X | 751 | 0.90378 | Truncation | 0.735378 | Truncation |
