# Supplementary material for: Two Rare Human Mitofusin 2 Mutations Alter Mitochondrial Dynamics and Induce Retinal and Cardiac Pathology in Drosophila
Source: PLoS One. 2012 Sep 5;7(9):e44296. doi: 10.1371/journal.pone.0044296 (PMC3434137; doi:10.1371/journal.pone.0044296)
Supplement: Table S2 — Pathological potential of possible HR1 amino acid mutations. (DOCX) [file pone.0044296.s003.docx]

**Table S2.** *Pathological potential of possible HR1 amino acid mutations.*

| **Ref**  **AA** | **Mut**  **AA** | **Ref DNA** | **Mut DNA** | **SIFT**  **score** | **SIFT**  **pred** | **Polyphen2**  **score** | **Polyphen2**  **pred** |
| --- | --- | --- | --- | --- | --- | --- | --- |
|  |  |  |  |  |  |  |  |
| **E391** | K | G | A | 0.96 | Damaging | 0 | Benign |
|  | Q | G | C | 0.91 | Tolerated | 0 | Benign |
|  | X | G | T | 0.902519 | Truncation | 0.730724 | Truncation |
|  | A | A | C | 0.93 | Tolerated | 0 | Benign |
|  | G | A | G | 0.98 | Damaging | 0 | Benign |
|  | V | A | T | 0.97 | Damaging | 0 | Benign |
|  | D | G | C | 0.98 | Damaging | 0 | Benign |
|  |  |  |  |  |  |  |  |
| **E392** | K | G | A | 0.96 | Damaging | 0.852 | Prob Damag |
|  | Q | G | C | 0.84 | Tolerated | 0.854 | Prob Damag |
|  | X | G | T | 0.903836 | Truncation | 0.735494 | Truncation |
|  | A | A | C | 0.96 | Damaging | 0.542 | Poss Damag |
|  | G | A | G | 0.99 | Damaging | 0.958 | Prob Damag |
|  | V | A | T | 0.89 | Tolerated | 0.053 | Benign |
|  | D | A | C | 0.67 | Tolerated | 0.937 | Prob Damag |
|  |  |  |  |  |  |  |  |
| **M393** | L | A | C | 0.7 | Tolerated | 0.095 | Benign |
|  | V | A | G | 0.81 | Tolerated | 0.036 | Benign |
|  |  |  |  |  |  |  |  |
|  | K | T | A | 0.35 | Tolerated | 0.001 | Benign |
|  | T | T | C | 0.6 | Tolerated | 0.001 | Benign |
|  | R | T | G | 0.66 | Tolerated | 0.047 | Benign |
|  | I | G | A | 0.88 | Tolerated | 0.161 | Poss Damag |
|  |  |  |  |  |  |  |  |
| **R394** | S | C | A | 0.99 | Damaging | 0.876 | Prob Damag |
|  | G | C | G | 0.99 | Damaging | 0.972 | Prob Damag |
|  | C | C | T | 0.91 | Tolerated | 0.02 | Benign |
|  | H | G | A | 0.99 | Damaging | 0.922 | Prob Damag |
|  | P | G | C | 0.99 | Damaging | 0.941 | Prob Damag |
|  | L | G | T | 0.98 | Damaging | 0.876 | Prob Damag |
|  |  |  |  |  |  |  |  |
| **E395** | K | G | A | 0.7 | Tolerated | 0.07 | Benign |
|  | Q | G | C | 0.92 | Tolerated | 0.73 | Poss Damag |
|  | X | G | T | 0.903836 | Truncation | 0.735494 | Truncation |
|  | A | A | C | 0.93 | Tolerated | 0.983 | Prob Damag |
|  | G | A | G | 0.97 | Damaging | 0.983 | Prob Damag |
|  | V | A | T | 0.98 | Damaging | 0.958 | Prob Damag |
|  | D | A | C | 0.79 | Tolerated | 0.937 | Prob Damag |
|  |  |  |  |  |  |  |  |
| **E396** | K | G | A | 0.48 | Tolerated | 0.486 | Poss Damag |
|  | Q | G | C | 0.7 | Tolerated | 0.707 | Poss Damag |
|  | X | G | T | 0.903836 | Truncation | 0.735493 | Truncation |
|  | A | A | C | 0.62 | Tolerated | 0.092 | Benign |
|  | G | A | G | 0.8 | Tolerated | 0.669 | Poss Damag |
|  | V | A | T | 0.83 | Tolerated | 0.482 | Poss Damag |
|  | D | G | C | 0.53 | Tolerated | 0.003 | Benign |
|  |  |  |  |  |  |  |  |
| **R397** | G | C | G | 0.95 | Tolerated | 0.712 | Poss Damag |
|  | W | C | T | 0.97 | Damaging | 0.854 | Prob Damag |
|  | Q | G | A | 0.74 | Tolerated | 0.003 | Benign |
|  | P | G | C | 0.93 | Tolerated | 0.778 | Poss Damag |
|  | L | G | T | 0 | Tolerated | 0.008 | Benign |
|  |  |  |  |  |  |  |  |
| **Q398** | K | C | A | 0.11 | Tolerated | 0 | Benign |
|  | E | C | G | 0.01 | Tolerated | 0.002 | Benign |
|  | X | C | T | 0.903778 | Truncation | 0.735314 | Truncation |
|  | P | A | C | 0.77 | Tolerated | 0.125 | Benign |
|  | R | A | G | 0.52 | Tolerated | 0.002 | Benign |
|  | L | A | T | 0.75 | Tolerated | 0.063 | Benign |
|  | H | A | C | 0.9 | Tolerated | 0.063 | Benign |
|  |  |  |  |  |  |  |  |
| **D399** | N | G | A | 0.9 | Tolerated | 0.011 | Benign |
|  | H | G | C | 0.99 | Damaging | 0.526 | Poss Damag |
|  | Y | G | T | 1 | Damaging | 0.864 | Prob Damag |
|  | A | A | C | 0.99 | Damaging | 0.526 | Poss Damag |
|  | G | A | G | 0.99 | Damaging | 0.338 | Poss Damag |
|  | V | A | T | 1 | Damaging | 0.713 | Poss Damag |
|  | E | C | A | 0.76 | Tolerated | 0.004 | Benign |
|  |  |  |  |  |  |  |  |
| **R400** | G | C | G | 0.97 | Damaging | 1 | Prob Damag |
|  | X | C | T | 0.897625 | Truncation | 0.722706 | Truncation |
|  | Q | G | A | 0.99 | Damaging | 0.999 | Prob Damag |
|  | P | G | C | 0.99 | Damaging | 1 | Prob Damag |
|  | L | G | T | 0.99 | Damaging | 1 | Prob Damag |
|  |  |  |  |  |  |  |  |
| **L401** | M | C | A | 0.99 | Damaging | 0.98 | Prob Damag |
|  | V | C | G | 0.99 | Damaging | 0.98 | Prob Damag |
|  | Q | T | A | 1 | Damaging | 0.995 | Prob Damag |
|  | P | T | C | 1 | Damaging | 0.998 | Prob Damag |
|  | R | T | G | 1 | Damaging | 0.995 | Prob Damag |
|  |  |  |  |  |  |  |  |
| **K402** | Q | A | C | 0.81 | Tolerated | 0 | Benign |
|  | E | A | G | 0.61 | Tolerated | 0 | Benign |
|  | X | A | T | 0.887151 | Truncation | 0.699504 | Truncation |
|  | T | A | C | 0.93 | Tolerated | 0 | Benign |
|  | R | A | G | 0.75 | Tolerated | 0 | Benign |
|  | I | A | T | 0.98 | Damaging | 0.003 | Benign |
|  | N | A | C | 0.94 | Tolerated | 0 | Benign |
|  |  |  |  |  |  |  |  |
| **F403** | I | T | A | 0.8 | Tolerated | 0.956 | Prob Damag |
|  | L | T | C | 0.66 | Tolerated | 0.956 | Prob Damag |
|  | V | T | G | 0.69 | Tolerated | 0.211 | Poss Damag |
|  | Y | T | A | 0.27 | Tolerated | 0.015 | Benign |
|  | S | T | C | 0.7 | Tolerated | 0.98 | Prob Damag |
|  | C | T | G | 0.91 | Tolerated | 0.991 | Prob Damag |
|  |  |  |  |  |  |  |  |
| **I404** | L | A | C | 0.9 | Tolerated | 0.077 | Benign |
|  | V | A | G | 0.07 | Tolerated | 0.003 | Benign |
|  | F | A | T | 0.98 | Damaging | 0.503 | Poss Damag |
|  | N | T | A | 0.99 | Damaging | 0.895 | Prob Damag |
|  | T | T | C | 0.33 | Tolerated | 0.027 | Benign |
|  | S | T | G | 0.99 | Damaging | 0.684 | Poss Damag |
|  | M | T | G | 0.98 | Damaging | 0.02 | Benign |
|  |  |  |  |  |  |  |  |
| **D405** | N | G | A | 0.98 | Damaging | 0.006 | Benign |
|  | H | G | C | 0.99 | Damaging | 0.024 | Benign |
|  | Y | G | T | 0.99 | Damaging | 0.125 | Benign |
|  | A | A | C | 0.95 | Tolerated | 0.024 | Benign |
|  | G | A | G | 0.98 | Damaging | 0.025 | Benign |
|  | V | A | T | 0.99 | Damaging | 0.025 | Benign |
|  |  |  |  |  |  |  |  |
| **K406** | Q | A | C | 0.82 | Tolerated | 0.16 | Poss Damag |
|  | E | A | G | 0.61 | Tolerated | 0.009 | Benign |
|  | X | A | T | 0.903758 | Truncation | 0.735333 | Truncation |
|  | T | A | C | 0.07 | Tolerated | 0.012 | Benign |
|  | R | A | G | 0.73 | Tolerated | 0.514 | Poss Damag |
|  | I | A | T | 0.96 | Damaging | 0.838 | Poss Damag |
|  | N | A | C | 0.14 | Tolerated | 0.002 | Benign |
|  |  |  |  |  |  |  |  |
| **Q407** | K | C | A | 0.98 | Damaging | 0.999 | Prob Damag |
|  | E | C | G | 0.99 | Damaging | 0.999 | Prob Damag |
|  | P | A | C | 1 | Damaging | 0.999 | Prob Damag |
|  | R | A | G | 0.89 | Tolerated | 0.999 | Prob Damag |
|  | L | A | T | 1 | Damaging | 0.999 | Prob Damag |
|  | H | G | C | 1 | Damaging | 0.999 | Prob Damag |
|  |  |  |  |  |  |  |  |
| **L408** | M | C | A | 0.51 | Tolerated | 0.007 | Benign |
|  | V | C | G | 0.99 | Damaging | 0.569 | Poss Damag |
|  | Q | T | A | 1 | Damaging | 0.966 | Prob Damag |
|  | P | T | C | 1 | Damaging | 0.966 | Prob Damag |
|  | R | T | G | 1 | Damaging | 0.966 | Prob Damag |
|  |  |  |  |  |  |  |  |
| **E409** | K | G | A | 0.41 | Tolerated | 0 | Benign |
|  | Q | G | C | 0.58 | Tolerated | 0.006 | Benign |
|  | X | G | T | 0.903836 | Truncation | 0.735493 | Truncation |
|  | A | A | C | 0.54 | Tolerated | 0.006 | Benign |
|  | G | A | G | 0.74 | Tolerated | 0.011 | Benign |
|  | V | A | T | 0.83 | Tolerated | 0.025 | Benign |
|  | D | G | C | 0.4 | Tolerated | 0 | Benign |
|  |  |  |  |  |  |  |  |
| **L410** | I | C | A | 0.65 | Tolerated | 0.009 | Benign |
|  | V | C | G | 0.77 | Tolerated | 0.16 | Poss Damag |
|  | F | C | T | 0.77 | Tolerated | 0.003 | Benign |
|  | H | T | A | 0.91 | Tolerated | 0.669 | Poss Damag |
|  | P | T | C | 0.94 | Tolerated | 0.903 | Prob Damag |
|  | R | T | G | 0.92 | Tolerated | 0.903 | Prob Damag |
|  |  |  |  |  |  |  |  |
| **L411** | M | T | A | 0.88 | Tolerated | 0.009 | Benign |
|  | V | T | G | 0.44 | Tolerated | 0.012 | Benign |
|  | X | T | A | 0.903691 | Truncation | 0.735194 | Truncation |
|  | S | T | C | 0.98 | Damaging | 0.482 | Poss Damag |
|  | W | T | G | 0.99 | Damaging | 0.838 | Poss Damag |
|  | F | G | C | 0.95 | Tolerated | 0.303 | Poss Damag |
|  |  |  |  |  |  |  |  |
| **A412** | T | G | A | 0 | Tolerated | 0 | Benign |
|  | P | G | C | 0.99 | Damaging | 0 | Benign |
|  | S | G | T | 0.99 | Damaging | 0 | Benign |
|  | D | C | A | 1 | Damaging | 0.001 | Benign |
|  | G | C | G | 1 | Damaging | 0.001 | Benign |
|  | V | C | T | 0.95 | Tolerated | 0 | Benign |
|  |  |  |  |  |  |  |  |
| **Q413** | K | C | A | 0.25 | Tolerated | 0.063 | Benign |
|  | E | C | G | 0.16 | Tolerated | 0 | Benign |
|  | X | C | T | 0.903795 | Truncation | 0.73512 | Truncation |
|  | P | A | C | 0.84 | Tolerated | 0.237 | Poss Damag |
|  | R | A | G | 0 | Tolerated | 0.063 | Benign |
|  | L | A | T | 0.56 | Tolerated | 0.002 | Benign |
|  | H | A | C | 0.9 | Tolerated | 0.063 | Benign |
|  |  |  |  |  |  |  |  |
| **D414** | N | G | A | 0.99 | Damaging | 0.474 | Poss Damag |
|  | H | G | C | 0.83 | Tolerated | 0.831 | Poss Damag |
|  | Y | G | T | 1 | Damaging | 0.899 | Prob Damag |
|  | A | A | C | 0.98 | Damaging | 0.777 | Poss Damag |
|  | G | A | G | 0.99 | Damaging | 0.777 | Poss Damag |
|  | V | A | T | 1 | Damaging | 0.777 | Poss Damag |
|  | E | C | A | 0.15 | Tolerated | 0.003 | Benign |
|  |  |  |  |  |  |  |  |
| **Y415** | N | T | A | 0.77 | Tolerated | 0.008 | Benign |
|  | H | T | C | 0.59 | Tolerated | 0.008 | Benign |
|  | D | T | G | 0.85 | Tolerated | 0.017 | Benign |
|  | S | A | C | 0.68 | Tolerated | 0 | Benign |
|  | C | A | G | 0.74 | Tolerated | 0 | Benign |
|  | F | A | T | 0.37 | Tolerated | 0 | Benign |
|  |  |  |  |  |  |  |  |
| **K416** | Q | A | C | 0.99 | Damaging | 0.293 | Poss Damag |
|  | E | A | G | 0.9 | Tolerated | 0.011 | Benign |
|  | T | A | C | 0.99 | Damaging | 0.777 | Poss Damag |
|  | R | A | G | 0.97 | Damaging | 0.011 | Benign |
|  | M | A | T | 1 | Damaging | 0.899 | Prob Damag |
|  | N | G | C | 0.99 | Damaging | 0.777 | Poss Damag |
|  |  |  |  |  |  |  |  |
| **L417** | M | C | A | 0.83 | Tolerated | 0 | Benign |
|  | V | C | G | 0.71 | Tolerated | 0 | Benign |
|  |  |  |  |  |  |  |  |
| **R418** | G | C | G | 1 | Damaging | 0.705 | Poss Damag |
|  | Q | G | A | 0.99 | Damaging | 0.322 | Poss Damag |
|  | P | G | C | 1 | Damaging | 0.378 | Poss Damag |
|  | L | G | T | 1 | Damaging | 0.705 | Poss Damag |
|  |  |  |  |  |  |  |  |
| **I419** | L | A | C | 0.88 | Tolerated | 0.99 | Prob Damag |
|  | V | A | G | 0.99 | Damaging | 0.99 | Prob Damag |
|  | F | A | T | 1 | Damaging | 0.998 | Prob Damag |
|  | N | T | A | 1 | Damaging | 0.999 | Prob Damag |
|  | T | T | C | 1 | Damaging | 0.998 | Prob Damag |
|  | S | T | G | 1 | Damaging | 0.999 | Prob Damag |
|  | M | T | G | 0.99 | Damaging | 0.995 | Prob Damag |
|  |  |  |  |  |  |  |  |
| **K420** | Q | A | C | 0.85 | Tolerated | 0.474 | Poss Damag |
|  | E | A | G | 0.91 | Tolerated | 0.47 | Poss Damag |
|  | T | A | C | 0.93 | Tolerated | 0.899 | Prob Damag |
|  | R | A | G | 0.57 | Tolerated | 0.003 | Benign |
|  | M | A | T | 0.98 | Damaging | 0.777 | Poss Damag |
|  | N | G | C | 0.94 | Tolerated | 0.777 | Poss Damag |
|  |  |  |  |  |  |  |  |
| **Q421** | K | C | A | 0.38 | Tolerated | 0.001 | Benign |
|  | E | C | G | 0.19 | Tolerated | 0 | Benign |
|  | P | A | C | 0.84 | Tolerated | 0.025 | Benign |
|  | R | A | G | 0.64 | Tolerated | 0.001 | Benign |
|  | L | A | T | 0.79 | Tolerated | 0.001 | Benign |
|  | H | G | C | 0.64 | Tolerated | 0 | Benign |
|  |  |  |  |  |  |  |  |
| **I422** | L | A | C | 0.53 | Tolerated | 0.036 | Benign |
|  | V | A | G | 0.59 | Tolerated | 0.003 | Benign |
|  | F | A | T | 0.98 | Damaging | 0.569 | Poss Damag |
|  | N | T | A | 1 | Damaging | 0.966 | Prob Damag |
|  | T | T | C | 0.99 | Damaging | 0.738 | Poss Damag |
|  | S | T | G | 1 | Damaging | 0.836 | Poss Damag |
|  | M | T | G | 0.34 | Tolerated | 0.026 | Benign |
|  |  |  |  |  |  |  |  |
| **T423** | P | A | C | 0.99 | Damaging | 0.958 | Prob Damag |
|  | A | A | G | 0.98 | Damaging | 0.73 | Poss Damag |
|  | S | A | T | 0.8 | Tolerated | 0.018 | Benign |
|  | K | C | A | 0.99 | Damaging | 0.852 | Prob Damag |
|  | R | C | G | 0.99 | Damaging | 0.983 | Prob Damag |
|  | M | C | T | 1 | Damaging | 0.987 | Prob Damag |
|  |  |  |  |  |  |  |  |
| **E424** | K | G | A | 0.89 | Tolerated | 0.006 | Benign |
|  | Q | G | C | 0.98 | Damaging | 0.168 | Poss Damag |
|  | A | A | C | 0.98 | Damaging | 0.031 | Benign |
|  | G | A | G | 0.99 | Damaging | 0.44 | Poss Damag |
|  | V | A | T | 0.99 | Damaging | 0.44 | Poss Damag |
|  | D | G | C | 0.92 | Tolerated | 0.001 | Benign |
|  |  |  |  |  |  |  |  |
| **E425** | K | G | A | 0.99 | Damaging | 0.569 | Poss Damag |
|  | Q | G | C | 0.99 | Damaging | 0.235 | Poss Damag |
|  | X | G | T | 0.903837 | Truncation | 0.735488 | Truncation |
|  | A | A | C | 0.99 | Damaging | 0.966 | Prob Damag |
|  | G | A | G | 0.98 | Damaging | 0.917 | Prob Damag |
|  | V | A | T | 1 | Damaging | 0.966 | Prob Damag |
|  | D | A | C | 0.91 | Damaging | 0.036 | Benign |
|  |  |  |  |  |  |  |  |
| **V426** | M | G | A | 1 | Damaging | 0.862 | Prob Damag |
|  | L | G | C | 1 | Damaging | 0.742 | Poss Damag |
|  | E | T | A | 1 | Damaging | 0.96 | Prob Damag |
|  | A | T | C | 1 | Damaging | 0.862 | Prob Damag |
|  | G | T | G | 1 | Damaging | 0.984 | Prob Damag |
|  |  |  |  |  |  |  |  |
| **E427** | K | G | A | 0.97 | Damaging | 0.687 | Poss Damag |
|  | Q | G | C | 0.97 | Damaging | 0.849 | Poss Damag |
|  | A | A | C | 0.85 | Tolerated | 0.077 | Benign |
|  | G | A | G | 0.98 | Damaging | 0.027 | Benign |
|  | V | A | T | 0.99 | Damaging | 0.797 | Poss Damag |
|  | D | G | C | 0.93 | Tolerated | 0.503 | Poss Damag |
|  |  |  |  |  |  |  |  |
| **R428** | G | A | G | 0.89 | Tolerated | 0.102 | Benign |
|  | W | A | T | 0.99 | Damaging | 0.692 | Poss Damag |
|  | K | G | A | 0.05 | Tolerated | 0 | Benign |
|  | T | G | C | 0.83 | Tolerated | 0 | Benign |
|  | M | G | T | 0.96 | Damaging | 0.011 | Benign |
|  | S | G | C | 0.82 | Tolerated | 0.001 | Benign |
|  |  |  |  |  |  |  |  |
| **Q429** | K | C | A | 0 | Tolerated | 0.003 | Benign |
|  | E | C | G | 0.99 | Damaging | 0.011 | Benign |
|  | P | A | C | 0.99 | Damaging | 0.777 | Poss Damag |
|  | R | A | G | 0.95 | Tolerated | 0.47 | Poss Damag |
|  | L | A | T | 0.99 | Damaging | 0.886 | Prob Damag |
|  | H | G | C | 0.99 | Damaging | 0.616 | Poss Damag |
|  |  |  |  |  |  |  |  |
| **V430** | M | G | A | 1 | Damaging | 0.98 | Prob Damag |
|  | L | G | C | 1 | Damaging | 0.98 | Prob Damag |
|  | E | T | A | 1 | Damaging | 0.995 | Prob Damag |
|  | A | T | C | 1 | Damaging | 0.995 | Prob Damag |
|  | G | T | G | 1 | Damaging | 0.998 | Prob Damag |
|  |  |  |  |  |  |  |  |
| **S431** | T | T | A | 0.99 | Damaging | 0.937 | Prob Damag |
|  | P | T | C | 0.99 | Damaging | 0.983 | Prob Damag |
|  | A | T | G | 0.66 | Tolerated | 0.387 | Poss Damag |
|  | W | C | G | 1 | Damaging | 0.998 | Prob Damag |
|  | L | C | T | 1 | Damaging | 0.983 | Prob Damag |
|  |  |  |  |  |  |  |  |
| **T432** | P | A | C | 0.84 | Tolerated | 0 | Benign |
|  | A | A | G | 0.7 | Tolerated | 0 | Benign |
|  | S | A | T | 0.15 | Tolerated | 0 | Benign |
|  |  |  |  |  |  |  |  |
| **A433** | T | G | A | 1 | Damaging | 0.036 | Benign |
|  | P | G | C | 1 | Damaging | 0.859 | Prob Damag |
|  | S | G | T | 1 | Damaging | 0.036 | Benign |
|  | E | C | A | 1 | Damaging | 0.738 | Poss Damag |
|  | G | C | G | 1 | Damaging | 0.741 | Poss Damag |
|  | V | C | T | 1 | Damaging | 0.569 | Poss Damag |
|  |  |  |  |  |  |  |  |
| **M434** | L | A | C | 0.59 | Tolerated | 0.538 | Poss Damag |
|  | V | A | G | 0.99 | Damaging | 0.988 | Prob Damag |
|  | K | T | A | 1 | Damaging | 0.998 | Prob Damag |
|  | T | T | C | 1 | Damaging | 0.998 | Prob Damag |
|  | R | T | G | 1 | Damaging | 0.998 | Prob Damag |
|  | I | G | A | 0.98 | Damaging | 0.974 | Prob Damag |
